# The diagnostic challenge of very early-onset enterocolitis in an infant with XIAP deficiency

**DOI:** 10.1186/s12887-015-0522-5

**Published:** 2015-12-15

**Authors:** Martina Girardelli, Serena Arrigo, Arrigo Barabino, Claudia Loganes, Giuseppe Morreale, Sergio Crovella, Alberto Tommasini, Anna Monica Bianco

**Affiliations:** Department of Advanced Diagnostic and Clinical Trials, Institute for Maternal and Child Health, IRCCS “Burlo Garofolo”, Trieste, Italy; Gastroenterology and Endoscopy Unit, G. Gaslini Children’s Hospital-IRCCS, Genoa, Italy; Department of Medical, Surgical and Health Sciences, University of Trieste, Trieste, Italy; Hematopoietic Stem Cell Transplantation Unit, Haematology-Oncology Department, G. Gaslini Children’s Research Institute, Genoa, Italy

**Keywords:** XIAP, Primary Immunodeficiency, Very early onset IBD, Crohn’s like, Intractable colitis, Periodic fever

## Abstract

**Background:**

Aggressive course and resistance to treatments usually characterize very early onset inflammatory bowel disease (VEO-IBD). Some VEO-IBD cases are due to monogenic immune defects and can benefit from hematopoietic stem cell transplantation (HSCT).

**Case presentation:**

We describe a Caucasian male baby who presented in the first months of life macrophage activation syndrome, followed by intractable colitis, recurrent episodes of fever and mild splenomegaly. After several immunological, genetic and clinical investigations, subsequently a therapeutic attempt with colectomy, analysis of VEO-IBD-associated genes, revealed a causative mutation in *XIAP*. The genetic diagnosis of a primary immune deficiency allowed curing the boy with hematopoietic stem cell transplantation.

**Conclusion:**

Our report, together with novel findings from recent literature, should contribute to increase awareness of monogenic immune defects as a cause of VEO-IBD. Comprehensive genetic analysis can allow a prompt diagnosis, resulting in the choice of effective treatments and sparing useless and damaging procedures.

## Background

Very early onset inflammatory bowel disease (VEO-IBD) is a rare and usually severe disorder, distinct from adult disease as regards extension, histopathology and treatment. A primary immunodeficiency (PID) may be the cause of the disease in a not negligible proportion of cases [1, 2]. Notably, gut inflammation can be the first and sole clinical manifestation of a PID for several years, while infections can develop later or remain underestimated. Indeed, IBD or IBD-like inflammation can be often the sole or the first manifestation of Chronic Granulomatous Disease (CGD) [3, 4], Wiskott Aldrich Disease [5], NEMO deficiency [6] or Polyendocrinopathy Enteropathy X-linked (IPEX) [7]. Recently also IL10R [8] and XIAP deficiencies [9] have been reported in children with early onset colitis, expanding the “universe of primary immunodeficiency” in IBD. Early onset IBD can be also presenting feature of autoinflammatory disorders, such as mevalonate kinase deficiency (MKD) [10, 11]. Moreover, functional studies have shown that VEO-IBD can occur in patients with *TTC7A* gene mutations, causing defects in the enterocytes and in T cells, giving rise to the development of a severe apoptotic enterocolitis [12]. Diagnosis of monogenic causes of early and very early onset-IBD is important in cases that could benefit from hematopoietic stem cell transplantation (HSCT). It is thus important to increase awareness of the possible monogenic etiology of VEO-IBD among pediatricians, promoting the development of reliable strategies for a prompt and thorough differential diagnosis.

## Case presentation

We report the case of a male baby, born to non-consanguineous healthy parents, who was hospitalized at 2 months of age with mucous and bloody diarrhea, fever and failure to thrive. The medical history revealed that he was admitted at the same hospital 1 month before because of high fever and hepatosplenomegaly that were attributed to a cytomegalovirus (CMV) infection with macrophage activation syndrome (MAS) (Table 1).

**Table 1:** Summary of clinical features and treatments

	1 month	2 – 3 months	11.5 months	15 months
Clinical features	High fever,	High fever	Intractable diarrhoea	Intractable diarrhoea, Vomit,
	Hepatomegaly,	Bloody and mucous diarrhoea		
			Recurrent fever	Recurrent fever
	Splenomegaly	Failure to thrive	Skin rash	
		Mild splenomegaly	Mild hepato-splenomegaly	
Haematological values	WBC 25.000/μl	WBC: 5.140/μl	WBC: 31.440/μl	WBC: 18.400/μl
	(N 38 %)	(N29%)	(N 55 %)	(N 62 %)
	CRP: 14,3 mg/L	CRP: 21 mg/L	CRP: 107 mg/L	CRP: 99 mg/L
	AST: 219 U/L	AST: 47 U/L		
	ALT: 327 U/L	ALT: 41 U/L		
	LDH 2300 U/L	Triglycerides: 2,39 mmol/L		
	Triglycerides: 3,08 mmol/L	Ferritin: 1.651 ng/ml		
	Ferritin: 18.000 ng/ml			
	IgA: 0,54 g/L			
	IgG: 5,67 g/L			
	IgM: 0,74 g/L			
	hypoalbumiemia			
Specialist investigations	Bone marrow smear: negative	Colonoscopy: inflammatory colitis with erosions and aphthae and increased cell apoptosis	Colonoscopy: Crohn’s-like colitis	
			EGDS normal	
		CMV negative in mucosa		
Virology	Blood CMV: 435.800 copies/ml	Blood CMV: negative		
Immunological evaluations	Normal Degranulation Assay		DHR test normal	
Genetic evaluations	PRF1, SAP, STXBP2 wild type	FOXP3: wild type	MVK: wild type	IL10, IL10RA, L10RB: wild type
				XIAP: mutate
Therapeutic interventions	Glucocorticoids, ganciclovir	Intravenous glucocorticoids, tacrolimus,	Glucocorticoids, Azathioprine, Adalimumab	Glucocorticoids, Adalimumab,
				TPN
		TPN	TPN	Antibiotics
Surgical intervention	none	none	Colectomy	HSCT

*DHR* dihydrorhodamine

*TPN* total parenteral nutrition

*HSCT* hematopoietic stem cell transplantation

*CRP* C Reactive Protein: normal values below 10 mg/L also the EGDS acronimous should be added in the legenEGDS Esophagogastroduodenoscopy

In spite of the absence of hemophagocytosis at the bone marrow smear, the main causes of familial lymphohistiocytosis were studied, yet with normal results. An antiviral therapy with ganciclovir, together with high dose dexamethasone, led to resolution of fever.

**Fig. 1:**
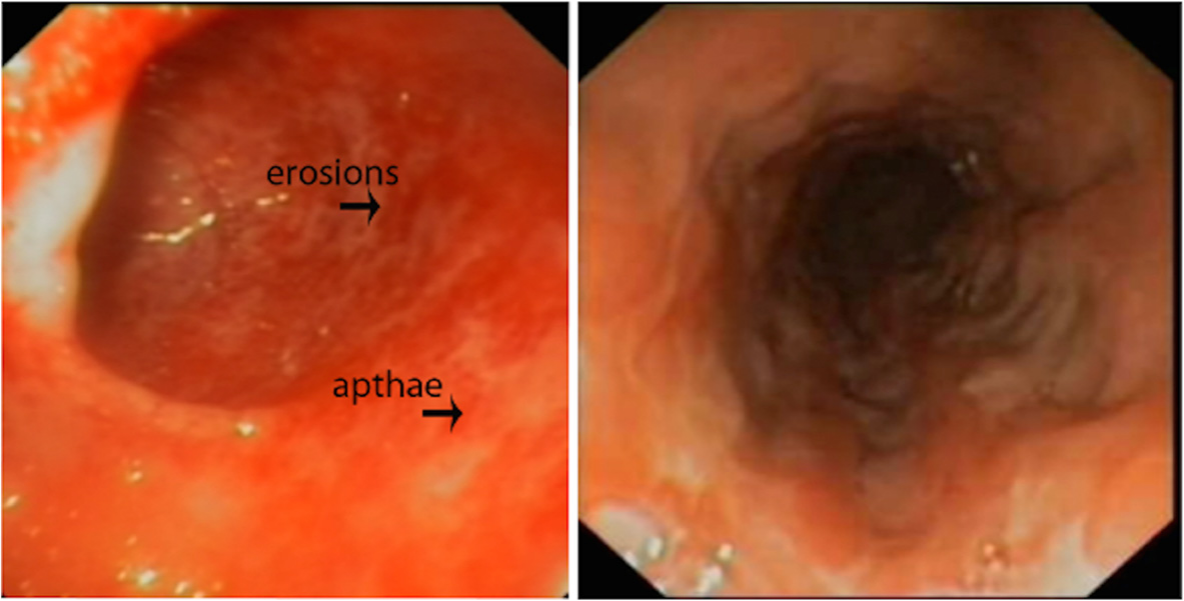
Image of colonoscopy investigation. In the pictures is possible to appreciate the colonoscopy features of the patient, in particular the erosions and apthae.

When the boy presented to our department, no sign of MAS was present and CMV was not detected in body fluids. A colonoscopy showed inflammatory colitis with erosions and aphthae (Fig. 1); the analysis of mucosal specimens revealed increased enterocyte apoptosis, leading to the suspicion of autoimmune enteropathy.

Genetic analysis of the *FOXP3* gene, responsible for IPEX, did not evidence any mutation. Total parenteral nutrition, tacrolimus and high dose steroids (10 mg/m^2^) were administered with good clinical and endoscopic response.

In the following months, after stopping steroids, in spite of continuing therapy with tacrolimus, the boy remained dependent on parenteral nutrition because of severe stunting. He developed several bouts of fever accompanied by increased acute phase reactants, worsening of diarrhea, skin rash and mild splenomegaly, but always not fulfilling the diagnostic criteria of MAS. Colonoscopy showed severe mucosal inflammation plus colon ulcerations, rectal and sigmoid erythema with fragile mucosa. In addiction edematous pseudopolypoid lesions and serpiginous ulcer covered by fibrin exudate have been observed from rectum to transverse colon. Ascending colon showed areas of reparative or scar tissue. Histologic examination confirmed the presence of a chronic inflammatory infiltrate in the lamina propria and submucosa with rare epithelioid granulomas. Terminal ileum was healthy.

Taking into account the novel clinical picture, MKD was suspected, but no mutation was detected in the *MVK* gene. Due to the presence of Crohn’s-like features, chronic granulomatous disease was also suspected, but the diagnosis was ruled out by normal results of dihydrorhodamine (DHR) flow cytometric assay.

A poor clinical response was obtained with standard dose glucocorticoids, azathioprine and, subsequently, with adalimumab (30 mg twice per month, subcutaneously). A transitory improvement was observed only after colectomy, however, recurrence of fever episodes persisted and the boy soon developed a Crohn’s-like ileitis.

**Fig. 2:**
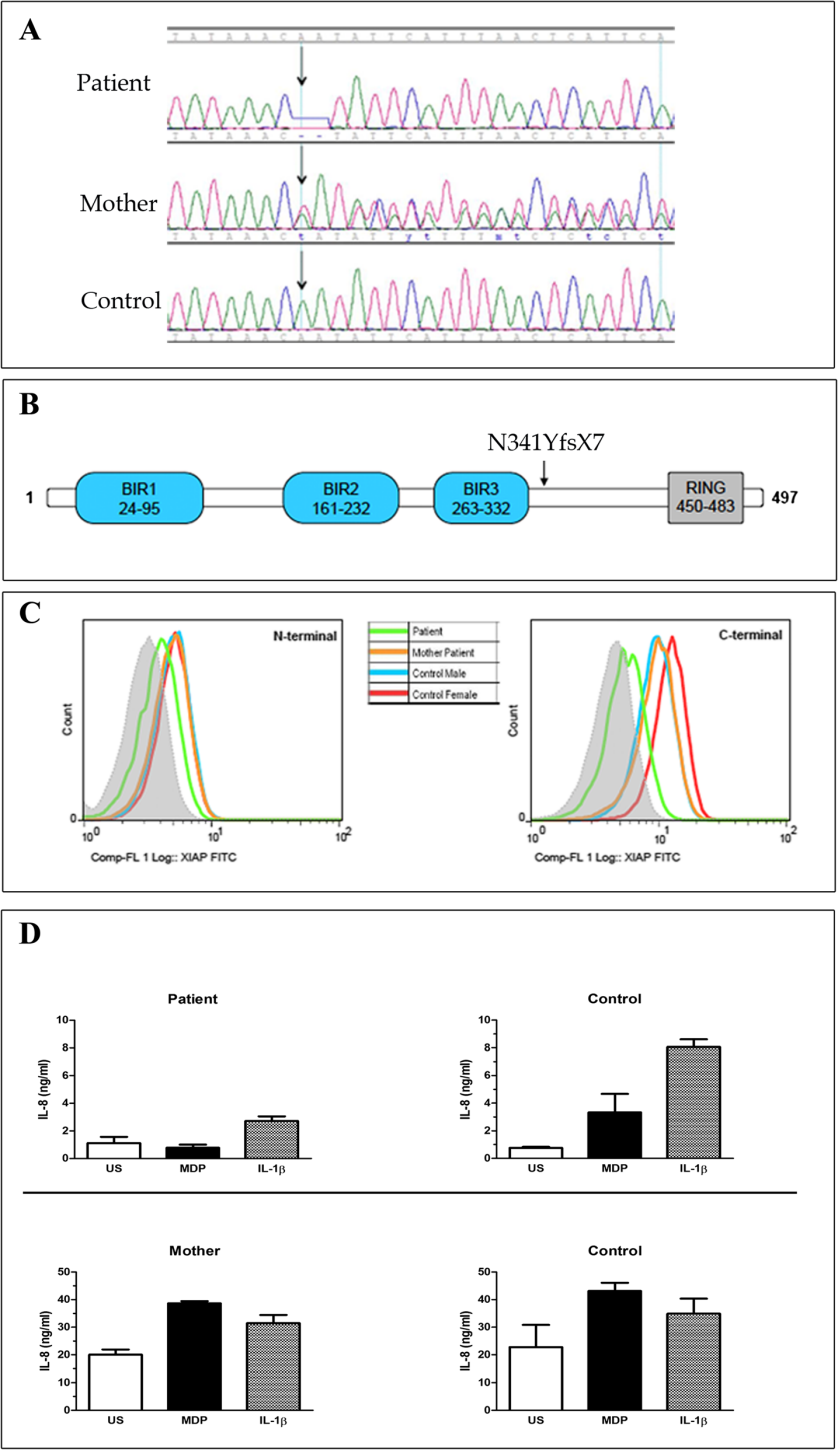
Electropherogram and functional test of *XIAP* mutation. **a** The Figure shows electopherograms of the mutation (c.1021_1022delAA) in exon 4 of *XIAP* in genomic DNA of patient, mother (heterozygous) and control (wild type). **b** Scheme of the protein structure of XIAP: BIR 1, 2, 3 and RING domains are shown. Black arrow indicates the localization of the mutation found in our patient. The mutation results in the substitution of the wild-type amino acids NIHLTHSLE with the mutant amino acids YSFNSFT until the stop codon and in the truncation of the protein at 347 amino acid of the 497 wild type protein. **c** Detection of the XIAP protein by flow cytometry on patient, his mother and in two healthy donors (male age related with patient and female controls age related with mother). The intracellular staining was performed with two different anti-human XIAP antibodies that recognize the N-terminal domain (amino acids 1-202) or the C-terminal domain (amino acid 268–426), respectively in the left and in the right side. The XIAP expression was evaluated on the CD45+ CD3+ cell gate. Grey area in the dashed line represents staining with secondary antibody alone. **d** NOD signalling pathway assay was performed testing patient, mother and age matched controls PBMCs unstimulated (US) or treated with IL-1β and MDP. The integrity of the pathway was measured using an IL-8 ELISA. PBMCs from our Crohn’s like patient were unable to induce the production of IL-8 after MDP stimulation, compared with wild-type controls and his mother, who carries in heterozygous the same mutation. The histograms report the mean of the values obtained by two different experiments.

**Fig. 3:**
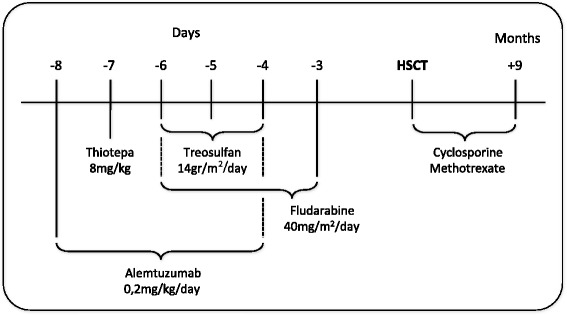
HSCT drug treatments. The figure illustrates the conditioning regimen administered to the patient before the HSCT and the combined drugs used for the prevention of graft-versus-host disease.

Further genetic analyses were thus performed, including the sequencing of Interleukin (IL)-10 Receptor (*IL10RA* and *IL10RB*), Interlukin-10 (*IL10*) and X-linked inhibitor of apoptosis (*XIAP*) genes. A deletion of two base-pairs was found in exon 4 of *XIAP*, causing a frameshift and a premature stop codon (RefSeq NM_001167, c.1021_1022delAA, p.N341YfsX7, Fig. 2a, b) [13]. The mutation was proven to be causative based on absent expression of the protein in T lymphocytes analyzed by flow cytometry with two anti-XIAP antibodies clones (clone48 BD Biosciences, cloneE-2 Santa Cruz Biotechnology). Since XIAP interacts with Nucleotide binding oligomerization domain 2 (NOD2), we also analyzed IL-8 production in response to muramyl dipeptide (MDP), revealing impaired signaling of this pathway (Fig. 2c, d). Based on the identification of *XIAP* mutation and the functional studies performed, the diagnosis of XIAP deficiency was made, so the patient underwent allogeneic-HSCT from a group A1 positive, CMV negative and EBV positive HLA-matched unrelated donor. He received peripheral stem cells (12,8x10^6^ CD34+ cells/kg) after a myeloablative-conditioning regimen as illustrated in Fig. 3. Nine days after transplantation, gastrointestinal bleeding with severe anaemia occurred. On day +15, micafungin was administered together with replacement of the central venous catheter (CVC) because of *Candida* sepsis. A CMV infection (+33 days) was successfully treated with ganciclovir and Foscavir. The patient developed an absolute neutrophil count above 500/μL on day +17 and a platelet counts above 50.000/μL on day +45. Six months after transplantation, a gradual improvement of appetite was observed, allowing the suspension of parenteral nutrition. The immunosuppressive therapy was interrupted after 9 months. Both upper endoscopy and ileoscopy were normal. On the last follow-up (+15 months), after temporary ileostomy reversal, the patient was found in good clinical conditions and no symptoms related to XIAP deficiency nor gastrointestinal problems were observed.

## Discussion

VEO-IBD can represent a serious diagnostic and therapeutic challenge. In our case, a monogenic cause for the disease was extensively searched because of complex clinical features such as a CMV-induced macrophage activation syndrome, recurrent fever, and intractable course of the disease.

A familial haemophagocytic lymphohistiocytosis was suspected and seemingly ruled out by functional and genetic analysis as well as by the good response to antiviral treatment. Afterwards, the association of early colitis with recurrent fever, rash and splenomegaly arouse the suspicion of Mevalonate Kinase Deficiency [10], yet genetic analysis excluded the presence of the disease.

Only some more months later, when disease relapsed in spite of colectomy, further genetic investigations were performed and the pathogenic deletion in *XIAP* gene identified.

The pathogenic role of XIAP in haemophagocytic lymphohistiocytosis is well documented [14, 15], and probably due to increased apoptosis of T cells during anti-viral responses [16, 17].

The deletion found in our patient, leading to a functional defect of NOD2 signaling, has been previously described in two brothers with recurrent lymphohistiocytosis but without intestinal inflammation [13]. Furthermore, other *XIAP* mutations have been described in cases with IBD [9, 18, 19]. Notably, mutations affecting BIR-2 domain of XIAP have been associated with impairment of NOD2 signaling [20] and IBD-like manifestations [19, 21]. Also Aguilar et al. have described a clinical overlap between Crohn’s disease and XIAP deficiency-associated IBD [22].

The *XIAP* mutation described here can thus explain both recurrent episodes of fever with mild splenomegaly, which likely represent incomplete bouts of lymphohistiocytosis, and intractable inflammatory colitis. In fact, XIAP deficiency is recognized as a highly heterogeneous disorder, whose expression probably depends upon the different type of mutation, environmental and infectious factors. Thus, although this is a serious primary immunodeficiency, the indication for HSCT is not always an easy issue, due to high transplantation related risks [23]. Nevertheless, recent studies show that idiopathic colitis with XIAP deficiency could be successfully treated with allo-HSCT using a specific conditioning regimen [24].

## Conclusion

We highlight the diagnostic challenge of VEO-IBD, with particular reference to possible monogenic defects of immunity. High-throughput genetic analysis can offer valuable option to cope with heterogeneity and severity of VEO-IBD. In our patient, an earlier genetic diagnosis could have promoted a timely HSCT, which could have been able to induce a complete remission, sparing colectomy. Although a sequential functional and genetic approach has been recommended [22], we suggest that functional investigations should not delay a promptly genetic analysis, to allow an early detection of rare monogenic disorders in children with VEO-IBD. We don’t have a long follow-up; nevertheless our results suggest that allo-HSCT is also an effective procedure for VEO-IBD.

## Consent

Written informed consent was obtained from the parents of the patient for publication of this Case report. A copy of the written consent is available for review by the Editor of this journal.

## Abbreviations

CMV: Cytomegalovirus
HSCT: Hematopoietic stem cell transplantation
MAS: Macrophage activation syndrome
NOD2: Nucleotide binding oligomerization domain 2
VEO-IBD: Very early onset inflammatory bowel disease
XIAP: X-linked inhibitor of apoptosis

## References

[1] Uhlig HH (2013). Monogenic diseases associated with intestinal inflammation: implications for the understanding of inflammatory bowel disease. Gut.

[2] Uhlig HH, Schwerd T, Koletzko S, Shah N, Kammermeier J, Elkadri A (2014). The diagnostic approach to monogenic very early onset inflammatory bowel disease. Gastroenterology.

[3] Jaggi P, Scherzer R, Knieper R, Mousa H, Prasad V (2012). Utility of screening for chronic granulomatous disease in patients with inflammatory bowel disease. J Clin Immunol.

[4] Freudenberg F, Wintergerst U, Roesen-Wolff A, Albert MH, Prell C, Strahm B (2010). Therapeutic strategy in p47-phox deficient chronic granulomatous disease presenting as inflammatory bowel disease. J Allergy Clin Immunol.

[5] Cannioto Z, Berti I, Martelossi S, Bruno I, Giurici N, Crovella S, Ventura A (2009). IBD and IBD mimicking enterocolitis in children younger than 2 years of age. Eur J Pediatr.

[6] Nenci A, Becker C, Wullaert A, Gareus R, van Loo G, Danese S (2007). Epithelial NEMO links innate immunity to chronic intestinal inflammation. Nature.

[7] Moraes-Vasconcelos D, Costa-Carvalho BT, Torgerson TR, Ochs HD (2008). Primary immune deficiency disorders presenting as autoimmune diseases: IPEX and APECED. J Clin Immunol.

[8] Glocker EO, Kotlarz D, Boztug K, Gertz EM, Schäffer AA, Noyan F (2009). Inflammatory bowel disease and mutations affecting the interleukin-10 receptor. N Engl J Med.

[9] Worthey EA, Mayer AN, Syverson GD, Helbling D, Bonacci BB, Decker B (2011). Making a definitive diagnosis: successful clinical application of whole exome sequencing in a child with intractable inflammatory bowel disease. Genet Med.

[10] Levy M, Arion A, Berrebi D, Cuisset L, Jeanne-Pasquier C, Bader-Meunier B (2013). Severe early-onset colitis revealing mevalonate kinase deficiency. Pediatrics.

[11] Oretti C, Barbi E, Marchetti F, Lepore L, Ventura A, D'Osualdo A (2006). Diagnostic challenge of hyper-IgD syndrome in four children with inflammatory gastrointestinal complaints. Scand J Gastroenterol.

[12] Avitzur Y, Guo C, Mastropaolo LA, Bahrami E, Chen H, Zhao Z (2014). Mutations in tetratricopeptide repeat domain 7A result in a severe form of very early onset inflammatory bowel disease. Gastroenterology.

[13] Yang X, Kanegane H, Nishida N, Imamura T, Hamamoto K, Miyashita R (2012). Clinical and genetic characteristics of XIAP deficiency in Japan. J Clin Immunol.

[14] Pachlopnik Schmid J, Canioni D, Moshous D, Touzot F, Mahlaoui N, Hauck F (2011). Clinical similarities and differences of patients with X-linked lymphoproliferative syndrome type 1 (XLP-1/SAP deficiency) versus type 2 (XLP-2/XIAP deficiency). Blood.

[15] Marsh RA, Madden L, Kitchen BJ, Mody R, McClimon B, Jordan MB (2010). XIAP deficiency: a unique primary immunodeficiency best classified as X-linked familial hemophagocytic lymphohistiocytosis and not as X-linked lymphoproliferative disease. Blood.

[16] Rumble JM, Oetjen KA, Stein PL, Schwartzberg PL, Moore BB, Duckett CS (2009). Phenotypic differences between mice deficient in XIAP and SAP, two factors targeted in X-linked lymphoproliferative syndrome (XLP). Cell Immunol.

[17] Rigaud S, Fondaneche MC, Lambert N, Pasquier B, Mateo V, Soulas P (2006). XIAP deficiency in humans causes an X-linked lymphoproliferative syndrome. Nature.

[18] Speckmann C, Lehmberg K, Albert MH, Damgaard RB, Fritsch M, Gyrd-Hansen M (2013). X-linked inhibitor of apoptosis (XIAP) deficiency: the spectrum of presenting manifestations beyond hemophagocytic lymphohistiocytosis. Clin Immunol.

[19] Zeissig Y, Petersen BS, Milutinovic S, Bosse E, Mayr G, Peuker K (2015). XIAP variants in male Crohn’s disease. Gut.

[20] Damgaard RB, Fiil BK, Speckmann C, Yabal M, zur Stadt U, Bekker-Jensen S (2013). Disease-causing mutations in the XIAP BIR2 domain impair NOD2-dependent immune signalling. EMBO Mol Med.

[21] Ammann S, Elling R, Gyrd-Hansen M, Dückers G, Bredius R, Burns SO (2014). A new functional assay for the diagnosis of X-linked inhibitor of apoptosis (XIAP) deficiency. Clin Exp Immunol.

[22] Aguilar C, Lenoir C, Lambert N, Bègue B, Brousse N, Canioni D (2014). Characterization of Crohn disease in X-linked inhibitor of apoptosis-deficient male patients and female symptomatic carriers. J Allergy Clin Immunol.

[23] Marsh RA, Rao K, Satwani P, Lehmberg K, Müller I, Li D (2013). Allogeneic hematopoi- etic cell transplantation for XIAP deficiency: An international survey reveals poor outcomes. Blood.

[24] Tsuma Y, Imamura T, Ichise E, Sakamoto K, Ouchi K, Osone S (2015). Successful treatment of idiopathic colitis related to XIAP deficiency with allo-HSCT using reduced-intensity conditioning. Pediatr Transplant.

